# Differences in gene expression profiles between individual islets from case #6213

**DOI:** 10.1186/1471-2105-15-S10-P18

**Published:** 2014-09-29

**Authors:** Nataliya I Lenchik, Hao Chen, Dorothy N Kakoola, Ivan C Gerling

**Affiliations:** 1 Department of Medicine, University of Tennessee Health Science Center, Memphis, TN 38104, USA; 2 Department of Pharmacology, University of Tennessee Health Science Center, Memphis, TN 38163, USA

## Background

In histological studies, nPOD investigators have reported substantial differences between individual islets from the same donor. The purpose of our study was to evaluate if there were substantial differences between individual islets also at the global gene expression level.

## Materials and methods

We chose an AAB+ donor pancreas (#6213) with insulin and glucagon staining islets that had already been the focus of studies by other nPOD investigators due to HLA hyper-expression and virus presence (by IHC, ISH and PCR). Human pancreas cryo-sections were shipped from the nPOD tissue bank. Slides were fixed, dried, and consecutive sections of each islet captured. Tissue was collected from a total of eight individual islets and RNA was immediately extracted from the tissue and stored at -80C. The quantity and integrity (RIN number) of total RNA was determined. RNA was amplified and Affymetrix Human Gene 2.0 ST arrays were used to obtain expression profiles.

## Results

The dataset for the eight samples was first filtered to exclude all probesets that did not reach a mean expression value of >4.0 (normalized expression value). We then analyzed each of the eight samples for “outlier genes” defined as genes whose expression value deviated by >4SD from the mean of the other seven samples. Furthermore we conducted hierarchical clustering on all expressed genes in the eight samples. The hierarchical clustering identified islet#7 as having an overall gene expression pattern deviating the most from the pattern in the other islets. This was also the sample with the largest number of outlier genes as defined above (>4SD). We subjected the list of outlier genes from each of the eight individual islets to a data-mining process to evaluate them for significant (p<10^-4^) enrichment for specific canonical pathways and upstream regulators (p<10^-4^). Only the list of outlier genes from islet#7 produced any canonical pathways or upstream regulators that passed this stringent statistical criterion. The three canonical pathways that were significantly enriched on the list of outlier genes from islet#7 were: “Antigen Presentation”, Actin Cytoskeleton Signaling” and “Virus Entry via Endocytotic Pathways”. The four most likely upstream regulators of the outlier gene expression patterns in islet#7 were TGF-beta1, lipopolysaccharide, TNF-alpha and Interferon-gamma.

## Conclusions

Our data suggest that there is a substantial variation in gene expression patterns within individual islets from the same donor. In our data one islet out of eight appeared to have a strong gene expression signature compatible with inflammation potentially induced by viral infection.

